# Prediction of non-muscle invasive bladder cancer recurrence using machine learning of quantitative nuclear features

**DOI:** 10.1038/s41379-021-00955-y

**Published:** 2021-10-29

**Authors:** Naoto Tokuyama, Akira Saito, Ryu Muraoka, Shuya Matsubara, Takeshi Hashimoto, Naoya Satake, Jun Matsubayashi, Toshitaka Nagao, Aashiq H. Mirza, Hans-Peter Graf, Eric Cosatto, Chin-Lee Wu, Masahiko Kuroda, Yoshio Ohno

**Affiliations:** 1 Department of Urology, Tokyo Medical University, Shinjuku-ku, Tokyo 160-0023 Japan; 2 Department of AI Applied Quantitative Clinical Science, Tokyo Medical University, Shinjuku-ku, Tokyo 160-8402 Japan; 3 Department of Molecular Pathology, Tokyo Medical University, Shinjuku-ku, Tokyo 160-8402 Japan; 4 Department of Anatomic Pathology, Tokyo Medical University, Shinjuku-ku, Tokyo 160-0023 Japan; 5 Department of Pharmacology, Weill Cornell Medicine, New York, NY 10065 USA; 6 Department of Machine Learning, NEC Labs America Inc., Princeton, NJ 08540 USA; 7 Department of Pathology, Massachusetts General Hospital, Boston, MA 02114 USA

**Keywords:** Bladder cancer, Bioinformatics

## Abstract

Non-muscle invasive bladder cancer (NMIBC) generally has a good prognosis; however, recurrence after transurethral resection (TUR), the standard primary treatment, is a major problem. Clinical management after TUR has been based on risk classification using clinicopathological factors, but these classifications are not complete. In this study, we attempted to predict early recurrence of NMIBC based on machine learning of quantitative morphological features. In general, structural, cellular, and nuclear atypia are evaluated to determine cancer atypia. However, since it is difficult to accurately quantify structural atypia from TUR specimens, in this study, we used only nuclear atypia and analyzed it using feature extraction followed by classification using Support Vector Machine and Random Forest machine learning algorithms. For the analysis, 125 patients diagnosed with NMIBC were used; data from 95 patients were randomly selected for the training set, and data from 30 patients were randomly selected for the test set. The results showed that the support vector machine-based model predicted recurrence within 2 years after TUR with a probability of 90% and the random forest-based model with probability of 86.7%. In the future, the system can be used to objectively predict NMIBC recurrence after TUR.

## Introduction

Bladder cancer is the ninth common malignant tumor worldwide^1^; it is clinically classified into non-muscle invasive bladder cancer (NMIBC) and muscle invasive bladder cancer (MIBC). Approximately 70% of bladder cancers are reported to be NMIBC at the time of initial diagnosis^2^. An important point is that the treatment strategy depends on the presence or absence of muscle layer invasion. Generally, NMIBC is considered to have a favorable prognosis. However, the rate of intravesical recurrence of NMIBC after transurethral resection of the bladder tumor (TURBT) is still as high as 30–50%^3^. To reduce the recurrence risk, bacillus Calmette-Guerin (BCG) therapy is recommended for high- and intermediate-risk categories. However, since BCG therapy is often associated with side effects such as hematuria, fever, and pain, its indications must be fully considered^4^. Thus, accurate evaluation of the recurrence risk is the most important factor in the management of NMIBC.

The current risk classification system provided by the American Urological Association is widely used. This classification system comprises clinical and pathological findings, such as the number of tumors, tumor size, recurrence history, depth of invasion, presence of carcinoma in situ, and tumor grade^5^. Other risk classification systems, including those defined by the European Association of Urology^6^, National Comprehensive Cancer Network^7^, and the Spanish Urological Club for Oncological Treatment^8^, comprise similar factors. However, even after using these risk classifications, previous reports have shown that a large number of patients relapse within 2 years after initial TURBT^9,10^. Conventional risk classifications are not complete to predict recurrence. Therefore, a novel risk assessment system from a new perspective is necessary.

In this study, we developed a novel system that uses artificial intelligence (AI) to determine the risk of recurrence. In particular, in developing this system, we focused on the characteristics of pathological specimens collected via TURBT. In other words, the T stage classification necessary for risk determination is determined by the presence or absence of muscle layers and the depth of invasion. However, these findings may not reflect the true lesion because they are affected by the sampling conditions^11^. Therefore, we deliberately did not capture information on structural atypia and invasive morphology of cells from pathological images obtained from TURBT specimens but used only information on cell nuclei, which is not affected by sampling conditions. As a result, we succeeded in constructing a system with very high prognostic accuracy only by extracting nuclear atypia. We believe that the results of this study will be a great asset for the extraction of nuclear atypia by AI using pathological images. Furthermore, it is expected that this system will be used for clinical applications in the future.

## Materials and methods

### Patients

We studied 162 patients who underwent TURBT between January 2012 and December 2019 at the Tokyo Medical University Hospital, with the approval of the hospital IRB (Institutional Review Board) based on the Declaration of Helsinki (approval number: SH3853). All cases were diagnosed as having NMIBC. They were stratified using the American Urological Association (AUA) risk criteria^5^. Regarding the AUA guideline, the high-risk cases were defined as tumors with HG (high grade)-pT1, HG-pTa with tumor size >3 cm or multifocal, or any CIS. Intermediate risk cases were defined as solitary tumors with LG (low grade)-Ta and tumor size >3 cm, multifocal tumors with LG-Ta, HG-Ta with tumor size ≤3 cm, or LG-T1. Initial and solitary tumors with LG-pTa and tumor size ≤3 cm were considered low-risk tumors. We defined standard treatment as TUR and adjuvant BCG or intravesical chemotherapy according to AUA risk criteria. We excluded Tis (carcinoma in situ) cases in this study because of the difficulty of complete tumor resection in the first transurethral resection (TUR) and the difference that BCG is performed therapeutic, not recurrence prevention. In addition, patients with variant histology, who were not followed for 2 years, who were diagnosed with intravesical recurrence after nephroureterectomy for upper urinary tract cancer, who had immediate total cystectomy after the first TURBT, who immediately received radiation therapy or systemic chemotherapy after initial TURBT were excluded. As a result, a total of 125 patients were finally included in this study. Recurrence was confirmed only when any of the lesions were pathologically confirmed as bladder cancer. Tumor was graded according to the 2016 World Health Organization classifications and was staged based on the UICC TNM classification 8th edition^12,13^.

### Follow-up and treatment

At our hospital, cystoscopy and urinary cytology were performed every 3 months for the first 2 years, every 6 months for the following 2 years, and once a year thereafter. All patients received immediate mitomycin C therapy within 24 h after TURBT. We performed repeated TUR for T1 cases. In patients with intermediate-risk or high-risk tumors, BCG therapy was considered. For BCG therapy, either 80 or 81 mg BCG was administered (Tokyo or Connaught strain) and repeated once a week for 6 or 8 consecutive weeks. No patient was treated with BCG maintenance therapy in the present study.

### Whole-slide scanning and image processing techniques

All hematoxylin and eosin-stained slides of the initial TURBT tissues were scanned at ×20 magnification using a whole-slide imaging scanner (Nanozoomer 2.0-HT slide scanner; Hamamatsu Corp., Hamamatsu, Shizuoka, Japan). On the scanned images, areas with viable tumor cells, high tumor cellularity, no necrosis, and no cautery effect are designated as regions of interest (ROI). One whole slide image size is ~1 GB. We manually selected ROIs from each whole slide image. ROIs were selected manually by a pathologist to identify the entire TURBT tissue tumor area without artifacts. The average number of ROIs for each case was seven; we selected at least 5 ROIs even in cases of small tumors, while in cases of large tumors, we selected a maximum of 15 ROIs to cover the cancerous area. Each ROI image size was 2048 × 2048 pixels, corresponding to 1 mm^2^.

### Quantitative nuclear feature extraction and ROI feature measurement

**Fig. 1:**
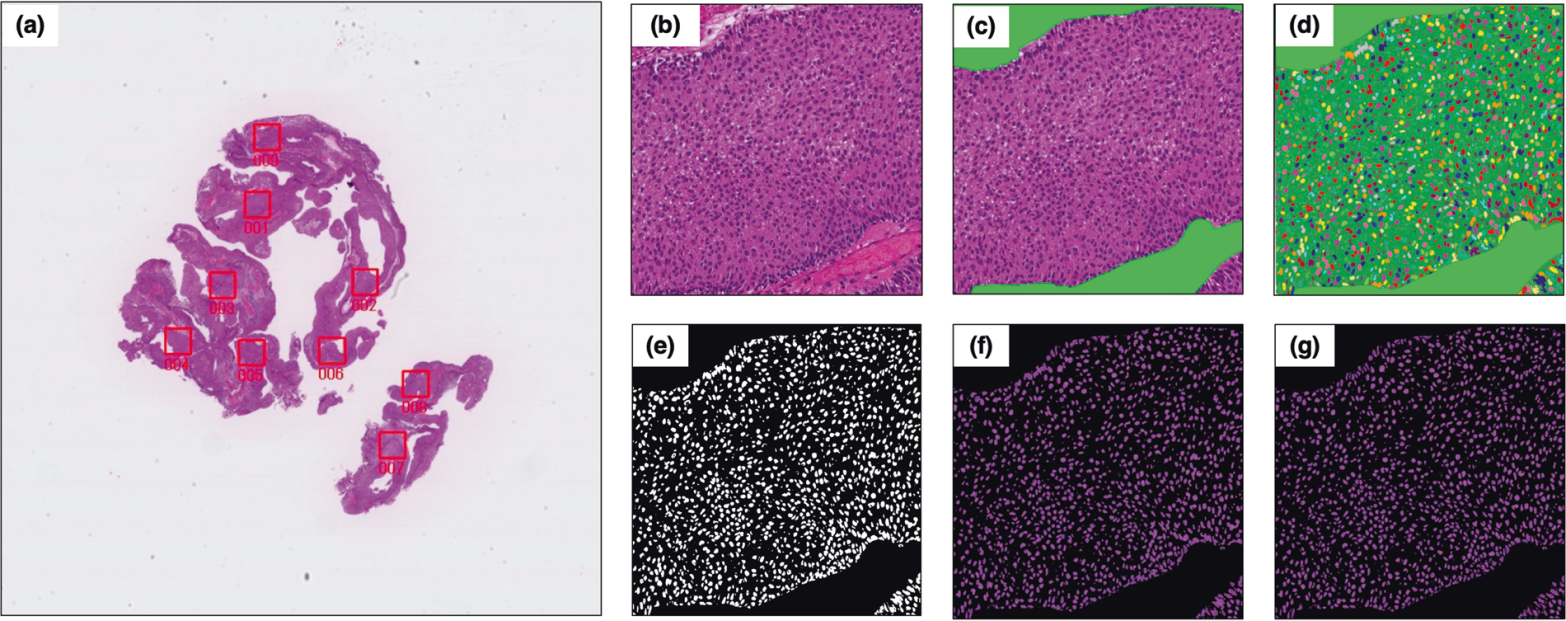
Extraction of nuclear morphological features. **a** Original hematoxylin and eosin-stained slide image. **b** Annotated region of interest (2048 × 2048 pixels). **c** Removal of no cancer cell area. **d** Automatic extraction of nuclei by “Ilastik.” **e** Creation of a masked image from (**d**). **f** Mask showing the inside of the nuclei area for measurements. **g** Separation of touching nuclei to precisely measure features for each nucleus.

A nuclear extraction process was performed for each ROI (Fig. 1). As a preparation step, the non-tumor area was manually masked. The computer automatically extracted nuclei from each ROI image. This process for nuclear extraction was performed using the free software program “Ilastik” (https://www.ilastik.org). Subsequently, we created masked images of nuclei inside area to prepare for measurements. A key step is to separate touching nuclei to ensure that feature measurements are performed on pixels belonging to a single nucleus. We created additional nuclei segmentation mask from detection model for individual nuclei using trained YOLO v3, a deep learning system useful for object detection^14^. This new nucleus mask image overlays the original extracted nuclear image (Fig. 1f), and a separate nucleus image is obtained (Fig. 1g). The process of this model is shown in Supplementary Fig 1. The first mask image using Ilastik focused on tracking the original contour line of the nucleus, even if it is connected. Additional mask using YOLOv3 focused on making a slit from independence of the nucleus. This double masking effectively extracted each nucleus.

Subsequently, “CellProfiler” (https://cellprofiler.org) was used to measure nuclear morphologic and texture features for each segmented nucleus. The following CellProfiler Modules were employed: MeasureObjectsSizeShape, MesureTexture, and MeasureObjectRadialDistribution. Details of the morphological features in CellProfiler can be found here: http://cellprofiler-manual.s3.amazonaws.com/CellProfiler-3.0.0/index.html. In addition, we employed a method called cell feature level co-occurrence matrix (CFLCM), which we previously reported^15^. CFLCM provides a way to evaluate the heterogeneity and pleomorphism of nuclei across an ROI image based on morphological and textural features of each nucleus. CFLCM features are derived from Haralick texture features based on the gray-level co-occurrence matrix.

### Machine learning (ML) methods, training, and model test

We selected support vector machine (SVM) and random forest (RF) as ML algorithms. Data were analyzed using the statistical software package R version 3.6.1 (R Project for Statistical Computing; https://www.r-project.org). We employed the package “e1071” on R to perform the SVM, and the package “randomForest” on R to perform the RF analysis. We used ROI features based on nuclear morphological information for machine learning and created a prediction model. The primary outcome was recurrence within 2 years. We randomly selected the test set to be 25% of the total patients. We used the average recurrence probabilities from each ROI, which were outputted by SVM and RF, as the case prediction result. The cases of equivalent probabilities were determined as undecidable. Finally, we checked the accuracy of correct classification in the test validation of the SVM model and the RF model. In the RF algorithm, we concurrently confirmed the out-of-bag error to evaluate the prediction performance of RF model.

## Results

### Patients characteristics and nuclear extraction

**Fig. 2:**
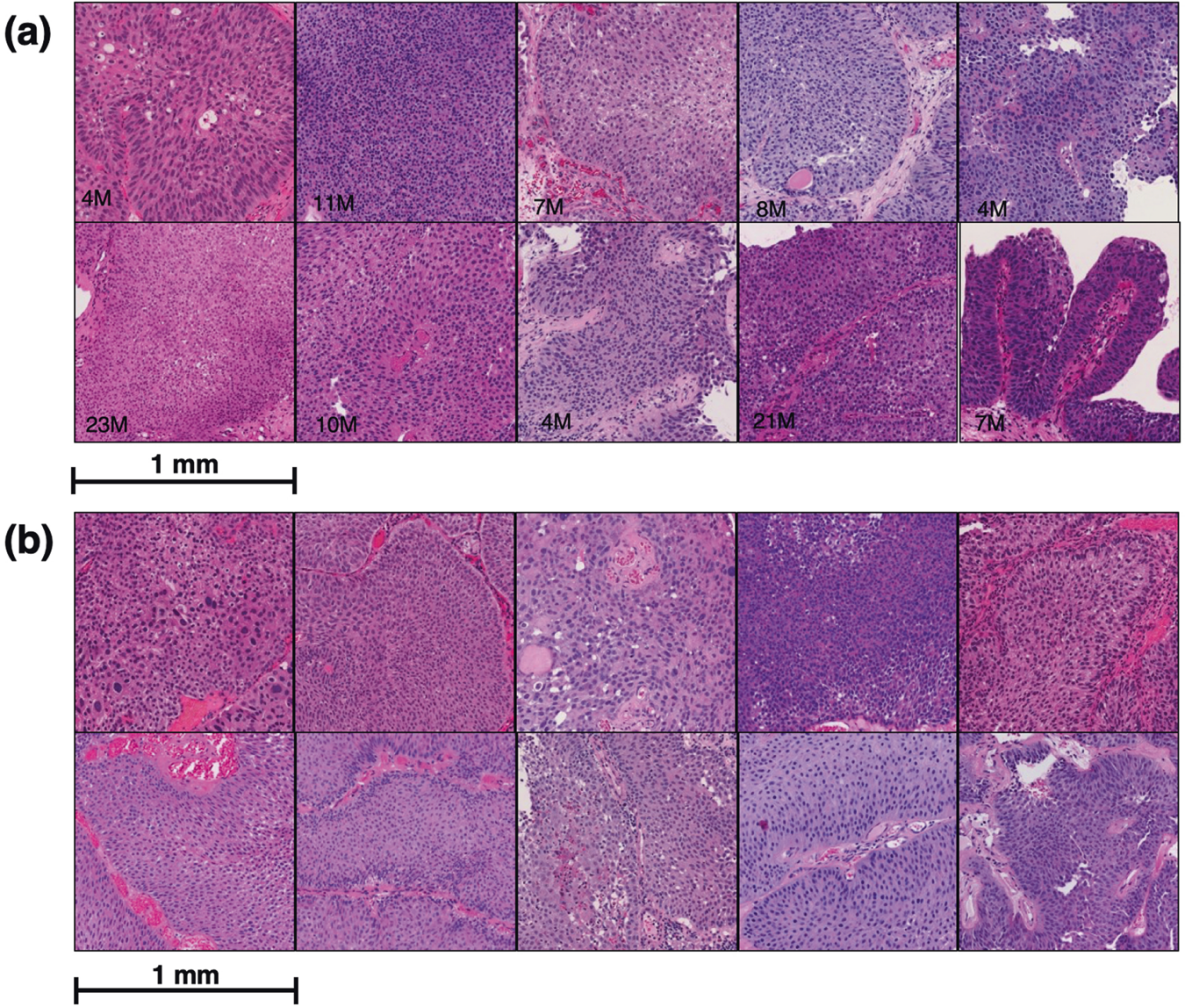
Some examples of the actual region of interest images. **a** Recurrence within 2 years, **b** no recurrence within 2 years. Recurrence period (M: months) is indicated in the lower-left corner of the recurrence images. It is not easy to distinguish the difference in nuclear morphology by visual inspection between two groups of histological images: recurrence and no recurrence.

Patients’ characteristics from the 125 cases are summarized in Table 1. The median observation period was 73 (range 24–192) months. Among the patients, 45 relapsed within 2 years after TUR, and 80 did not. A total of 216 whole slide images were acquired from all cases. Then, a total of 877 ROI images of the tumor area were acquired from those cases. We have indicated some examples of actual ROI images of each group in Fig. 2. Image processing was performed (see the “Methods” section for details) to extract quantitative nuclear features, and a total of 1008,502 nuclei were delineated.

**Table 1:** Patient characteristics.

Characteristics	Total *N* = 125	Recurrence within 2-years *N* = 45	Recurrence free within 2-years *N* = 80
Age (median, range)	71 (29–94)	73.5 (49–94)	70 (29–93)
Sex (male), *n*%	91 (73%)	37 (82%)	54 (68%)
Grade^a^			
Low grade	51	14	37
High grade	74	31	43
T stage			
a	65	22	43
1	60	23	37
Tumor number			
1	75	24	51
2	21	7	14
>3	29	14	15
Tumor size (cm)			
<3	103	38	65
>3	22	7	15
CIS			
−	112	40	72
+	13	5	8
Tumor shape			
Papillary	115	41	74
Non-papillary	10	4	6
BCG			
−	59	24	35
+	66	21	45
AUA risk stratification			
Low	31	10	21
Intermediate	29	9	20
High	65	26	39

*CIS* carcinoma in situ, *BCG* bacillus Calmette-Guerin, *AUA* American Urological Association.

^a^WHO 2016 classifications.

### Characteristics of nuclear morphological features

**Fig. 3:**
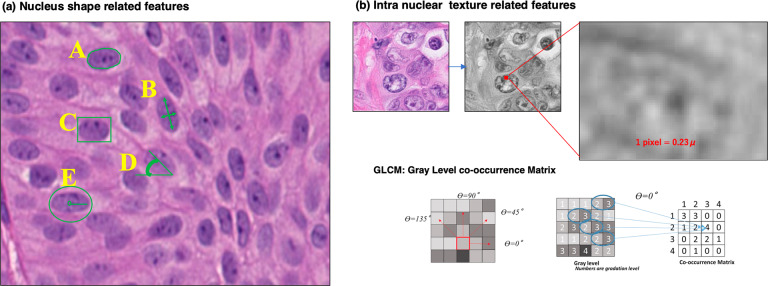
Illustration of the nucleus morphological features measured in this study. **a** Nucleus shape-related features: A, from the nucleus contour line (green), we obtained the nucleus area, perimeter, and roundness (4*π*Area/Perimeter^2^); B, long- and short-axis length and ratio (another measure of roundness); C, solidity (area/bounding-box size); D, orientation (angle between the long axis and horizontal axis); E, average radius. **b** Nuclear texture-related features (second angular moment, homogeneity, entropy, etc.) that indicate the texture of chromatin measured using the gray level co-occurrence matrix method with Haralick texture features, from which our cell feature level co-occurrence matrix features are derived.

Then, we extracted 79 quantitative morphological features of each identified nucleus using CellProfiler (https://cellprofiler.org). This morphological information can be classified into two major categories (Fig. 3). The first group comprises 27 features related to the shape of the nucleus, such as size, contour length, major axis length, roundness, solidity, and eccentricity. The other group consisted of 52 intranuclear texture-related features (second angular moment, homogeneity, entropy, etc.) (Supplementary Table 1). From these 79 nuclear features, we obtained ROI features using CFLCM and acquired a total of 960 features for each ROI.

### SVM model predict recurrence

**Fig. 4:**
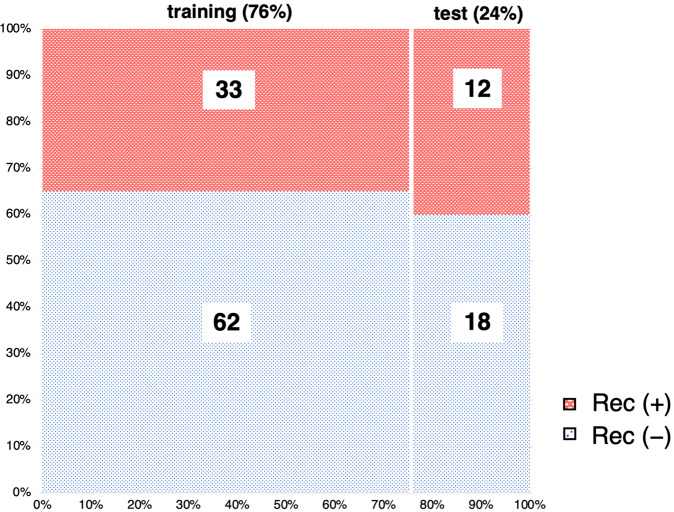
Data sets were divided into training and test sets. We randomly selected the 30 test sets to be ~25% of total cases. The vertical axis showed the percentage of each group, and the horizontal axis showed the rate of training and test sets. Recurrence within 2 years: Rec (+) was indicated by red, no recurrence within 2 years, and Rec (−) by blue.

To create a recurrence prediction model using ROI features, we employed the SVM as ML algorithm. The data set was randomly divided into 95 training and 30 test cases; this is shown in Fig. 4. Training cases were used to optimize the model, and test cases were used to check the accuracy of the classification. Classification of the ROI of each case into recurrence within 2 years and no recurrence within 2 years using the SVM model training showed an accuracy of 100% (Table 2a). The SVM model classifier was verified using test sets; the accuracy of the correct classification of the ROIs was 83.8% (Table 2b). Aggregating the results of each ROI to the cases resulted in 90% probability of correct classification (Table 2c). There were three incorrect discriminations (Supplementary Table 2, test cases 17, 22, and 23) in the model test. One case of Rec (−) was undecidable. Two cases of Rec (−) were incorrectly discriminated as Rec (+). Supplementary Table 3 shows the top 20 morphological features with high contributions to recurrence and non-recurrence in the SVM model. The features with high weights contribute the most to the classification.

**Table 2:** Results of support vector machine (SVM) model validation in test cases.

		Prediction		
		Rec (+)	Rec (−)	Total
(a) Trainig set result				
Accuracy: 100%				
Truth	Rec (+)	328	0	328
	Rec (−)	0	370	370
	Total	328	370	698
(b) Test set prediction result (ROI based)				
Accuracy: 83.8%				
Truth	Rec (+)	72	10	82
	Rec (−)	19	78	97
	Total	91	88	179
(c) Test set prediction result (case based)				
Accuracy: 90%				
Truth	Rec (+)	12	0	12
	Rec (−)	3	15	18
	Total	15	15	30

*Rec* recurrence within 2-years, *ROI* region of interest.

### RF model predict recurrence

We also implemented an analysis using another basic ML algorithm, RF to investigate which type of ML algorithm was optimal in this study. RF model training indicated an accuracy of 100% (Table 3a). We performed RF model verification using test sets; the accuracy of correct ROI-based classification was 74.9% (Table 3b). By contrast, the out-of-bag estimate of error rate was 15.6%, which indicated the validity of the RF model performance to ROI discrimination (Supplementary Table 4). Aggregated case-based classification resulted in 86.7% accuracy of correct classification for the test set (Table 3c). There were four errors (Supplementary Table 2, test cases 11, 17, 22, and 23) in the RF model test. Four cases of Rec (−) were incorrectly discriminated as Rec (+).

**Table 3:** Results of random forest (RF) model validation in test cases.

		Prediction		
		Rec (+)	Rec (−)	Total
(a) Trainig set result				
Accuracy: 100%				
Truth	Rec (+)	328	0	328
	Rec (−)	0	370	370
	Total	328	370	698
(b) Test set prediction result (ROI based)				
Accuracy: 74.9%				
Truth	Rec (+)	66	16	82
	Rec (−)	29	68	97
	Total	95	84	179
(c) Test set prediction result (case based)				
Accuracy: 86.7%				
Truth	Rec (+)	12	0	12
	Rec (−)	4	14	18
	Total	16	14	30

*Rec* recurrence within 2-years, *ROI* region of interest.

In ROI image classification, the accuracy was lower than that of the SVM model. However, for case prediction, we confirmed a comparable accuracy between the two prediction models.

## Discussion

Currently, AI based on digital pathology images is being used for diagnosis, morphological classification, and prognosis prediction of various cancers^16–18^. We have also developed prognostic systems for several cancers using ML and deep learning and have confirmed their usefulness^19,20^. Although it is very promising to create objective and accurate predictions from digital imaging data in various cancers, no previous studies have used AI on digital pathology images to predict the prognosis of NMIBC. The presumed reason is that many pathological specimens of bladder cancer are collected by TUR; specimens collected by TUR are different from surgical materials, and the tissue is significantly degenerated at the time of collection. In addition, the fragmented nature of the specimens makes it difficult to determine the structural atypia of the tumor. Therefore, in this study, we attempted to predict the prognosis of tumors using only nuclear morphological and textural information as an unprecedented method. As a result, we constructed a new classification system that does not depend on other clinical information. This acquisition of nuclear features is expected to significantly contribute to AI pathology in other carcinomas and diseases in the future.

In this study, two standard ML algorithms, SVM and RF, were evaluated to predict prognosis using only nuclear information. SVM and RF are well suited when the sample size is relatively small. We carefully avoided overfitting situations by using out-of-bag evaluation. We found that both SVM and RF models were able to classify a test set as either recurring within 2 years or not recurring within 2 years with significant accuracy. Interestingly, most of the errors were misclassified by both SVM and RF models. The misclassification was that a patient with Rec (−) was judged as Rec (+) (Supplementary Table 2, test cases 11, 17, 22, and 23). In fact, three of these patients had recurrence 3 years after TUR. In addition, one case recurred at 41 months after the first TUR. These results indicate that AI can accurately predict early recurrence by using a large number of features, even with only morphological and textural information of nuclear atypia. The fact that the SVM and RF models yielded similar results suggests that the morphological information of nuclear atypia is highly discriminative.

This model will be a new minimally invasive prediction model that provides information from the aspect of cancer morphology, which is completely different from the conventional risk classification (Supplementary Fig. 2). A weakness of the current risk classification is the heterogeneity of tumors belonging to intermediate-risk categories^21^. We believe that this analysis of nuclear features can be used as an auxiliary tool to improve the current risk classification. In the future, we need to investigate how other morphological information can be processed to improve the accuracy of AI-based prognostic prediction or how the number of cases can be increased to improve the accuracy. In addition, to avoid selection bias, it would be ideal to construct an automatic acquisition of ROIs for total slide images. As another limitation, to avoid bias, we did not include cases that received BCG maintenance therapy which significantly reduce recurrence rates. This might limit the generalizability of the results. However, the results indicate reasonable accuracy for recurrence prediction from the initial TUR tissue image. Henceforth, this method is expected to be applied to predict progression to muscle-invasive diseases, efficacy of intravesical injection therapy, and long-term prognosis.

In conclusion, our study demonstrated the usefulness of quantitative nuclear morphological information of cancer cells obtained using digital pathologic analysis in NMIBC patients, which we used to develop a novel recurrence risk prediction model. This model must contribute to the future development of ML models in NMIBC.

## Supplementary information

Supplementary Material

## Data Availability

The datasets used and analyzed during the current study are available from the corresponding author on reasonable request.
