# Supplementary material for: Prediction of non-muscle invasive bladder cancer recurrence using machine learning of quantitative nuclear features
Source: Mod Pathol. 2021 Oct 29;35(4):533–8. doi: 10.1038/s41379-021-00955-y (PMC8964412; doi:10.1038/s41379-021-00955-y)

**Supplementary Table 1. the list of nuclear features in this study**

| Feature category | Nuclear feature name from Cellprofiler |
|---|---|
| Nucleus shape related features | Area |
| | Compactness |
| | Eccentricity |
| | Extent |
| | FormFactor |
| | MajorAxisLength |
| | MaxFeretDiameter |
| | MaximumRadius |
| | MeanRadius |
| | MedianRadius |
| | MinFeretDiameter |
| | MinorAxisLength |
| | Orientation |
| | Perimeter |
| | Solidity |
| | Nuclei_RadialDistribution_FracAtD_1of4 |
| | Nuclei_RadialDistribution_FracAtD_2of4 |
| | Nuclei_RadialDistribution_FracAtD_3of4 |
| | Nuclei_RadialDistribution_FracAtD_4of4 |
| | Nuclei_RadialDistribution_MeanFrac_1of4 |
| | Nuclei_RadialDistribution_MeanFrac_2of4 |
| | Nuclei_RadialDistribution_MeanFrac_3of4 |
| | Nuclei_RadialDistribution_MeanFrac_4of4 |
| | Nuclei_RadialDistribution_RadialCV_1of4 |
| | Nuclei_RadialDistribution_RadialCV_2of4 |
| | Nuclei_RadialDistribution_RadialCV_3of4 |
| | Nuclei_RadialDistribution_RadialCV_4of4 |
| Intra nuclear texture related features | AngularSecondMoment_0 |
| | AngularSecondMoment_135 |
| | AngularSecondMoment_45 |
| | AngularSecondMoment_90 |
| | Contrast_0 |
| | Contrast_135 |
| | Contrast_45 |
| | Contrast_90 |
| | Correlation_0 |
| | Correlation_135 |
| | Correlation_45 |
| | Correlation_90 |
| | DifferenceEntropy_0 |
| | DifferenceEntropy_135 |
| | DifferenceEntropy_45 |
| | DifferenceEntropy_90 |
| | DifferenceVarianc_0 |
| | DifferenceVariance_135 |
| | DifferenceVariance_45 |
| | DifferenceVariance_90 |
| | Entropy_0 |
| | Entropy_135 |
| | Entropy_45 |
| | Entropy_90 |
| | InfoMeas_0 |
| | InfoMeas_135 |
| | InfoMeas_45 |
| | InfoMeas_90 |
| | InfoMeas2_0 |
| | InfoMeas2_135 |
| | InfoMeas2_45 |
| | InfoMeas2_90 |
| | InverseDifferenceMoment_0 |
| | InverseDifferenceMoment_135 |
| | InverseDifferenceMoment_45 |
| | InverseDifferenceMoment_90 |
| | SumAverage_0 |
| | SumAverage_135 |
| | SumAverage_45 |
| | SumAverage_90 |
| | SumEntropy_0 |
| | SumEntropy_135 |
| | SumEntropy_45 |
| | SumEntropy_90 |
| | SumVariance_0 |
| | SumVariance_135 |
| | SumVariance_45 |
| | SumVariance_90 |
| | Variance_Gray_0 |
| | Variance_135 |
| | Variance_45 |
| | Variance_90 |

Cellprofiler features
http://cellprofiler-manual.s3.amazonaws.com/CellProfiler-3.0.0/modules/measurement.html

**Supplementary Table 2. The results of probability to each group calculated by SVM and RF models in the validation.**

**(a) SVM-based model**

| Case | Truth Group | Prediction Group | Rec(+) probability | Rec(-) probability |
|---|---|---|---|---|
| 1 | – | – | 0.04 | 0.96 |
| 2 | + | + | 0.63 | 0.37 |
| 3 | – | – | 0.19 | 0.81 |
| 4 | – | – | 0.11 | 0.89 |
| 5 | + | + | 0.73 | 0.27 |
| 6 | – | – | 0.17 | 0.83 |
| 7 | – | – | 0.21 | 0.79 |
| 8 | – | – | 0.25 | 0.75 |
| 9 | + | + | 0.69 | 0.31 |
| 10 | – | – | 0.11 | 0.89 |
| 11 | – | – | 0.47 | 0.53 |
| 12 | + | + | 0.66 | 0.34 |
| 13 | + | + | 0.59 | 0.41 |
| 14 | + | + | 0.90 | 0.10 |
| 15 | + | + | 0.64 | 0.36 |
| 16 | + | + | 0.91 | 0.09 |
| 17 | – | + | 0.63 | 0.37 |
| 18 | + | + | 0.71 | 0.29 |
| 19 | + | + | 0.73 | 0.27 |
| 20 | + | + | 0.82 | 0.18 |
| 21 | + | + | 0.72 | 0.28 |
| 22 | – | + / – | 0.50 | 0.50 |
| 23 | – | + | 0.55 | 0.45 |
| 24 | – | – | 0.37 | 0.63 |
| 25 | – | – | 0.37 | 0.63 |
| 26 | – | – | 0.28 | 0.72 |
| 27 | – | – | 0.19 | 0.81 |
| 28 | – | – | 0.31 | 0.69 |
| 29 | – | – | 0.23 | 0.77 |
| 30 | – | – | 0.28 | 0.72 |

**(b) RF-based model**

| Case | Truth Group | Prediction Group | Rec(+) probability | Rec(-) probability |
|---|---|---|---|---|
| 1 | – | – | 0.37 | 0.63 |
| 2 | + | + | 0.62 | 0.38 |
| 3 | – | – | 0.36 | 0.64 |
| 4 | – | – | 0.36 | 0.64 |
| 5 | + | + | 0.61 | 0.39 |
| 6 | – | – | 0.32 | 0.68 |
| 7 | – | – | 0.24 | 0.76 |
| 8 | – | – | 0.26 | 0.74 |
| 9 | + | + | 0.72 | 0.28 |
| 10 | – | – | 0.29 | 0.71 |
| 11 | – | + | 0.53 | 0.47 |
| 12 | + | + | 0.64 | 0.36 |
| 13 | + | + | 0.67 | 0.33 |
| 14 | + | + | 0.74 | 0.26 |
| 15 | + | + | 0.64 | 0.36 |
| 16 | + | + | 0.76 | 0.24 |
| 17 | – | + | 0.73 | 0.27 |
| 18 | + | + | 0.63 | 0.37 |
| 19 | + | + | 0.71 | 0.29 |
| 20 | + | + | 0.74 | 0.26 |
| 21 | + | + | 0.62 | 0.38 |
| 22 | – | + | 0.60 | 0.40 |
| 23 | – | + | 0.70 | 0.30 |
| 24 | – | – | 0.36 | 0.64 |
| 25 | – | – | 0.20 | 0.80 |
| 26 | – | – | 0.41 | 0.59 |
| 27 | – | – | 0.29 | 0.71 |
| 28 | – | – | 0.33 | 0.67 |
| 29 | – | – | 0.33 | 0.67 |
| 30 | – | – | 0.29 | 0.71 |

Abbreviations: Rec, recurrence within 2-years; SVM, support vector machine; RF, random forest
The cases of misjudgment and undecidable are indicated in gray color.

**Supplementary Table 3. The top 20 morphological features with highly contributions to recurrence and non-recurrence in the SVM model.**

**(A) Top 20 of highly contributed features to Recurrence**

| A | B | C | D | E | F | G |
|---|---|---|---|---|---|---|
| Feature No. | Feature Name | Feature group | Method | Neighborhood | Haralick Feature | Weight |
| F002 | Area | 1 | Stdev | NI | NI | 43.918 |
| F001 | Area | 1 | Average | NI | NI | 33.486 |
| F707 | Entropy_max | 3 | ROI | Nearest | Contrast | 8.628 |
| F284 | MeanRadius | 2 | Case | Circle | Contrast | 8.265 |
| F269 | MeanRadius | 2 | Case | Nearest | Contrast | 7.945 |
| F119 | Extent | 2 | Group | Circle | Contrast | 6.235 |
| F616 | Correlation_max | 3 | Group | Nearest | Contrast | 5.878 |
| F717 | Entropy_max | 3 | Case | Nearest | Contrast | 5.69 |
| F712 | Entropy_max | 3 | Group | Nearest | Contrast | 5.649 |
| F631 | Correlation_max | 3 | Group | Circle | Contrast | 5.401 |
| F867 | SumEntropy_max | 3 | ROI | Circle | Contrast | 5.084 |
| F124 | Extent | 2 | Case | Nearest | Contrast | 4.942 |
| F296 | MedianRadius | 2 | Group | Nearest | Contrast | 4.926 |
| F520 | MeanFrac_ratio | 2 | Group | Nearest | Contrast | 4.78 |
| F168 | MajorAxisLength | 2 | Group | Nearest | Contrast | 4.518 |
| F264 | MeanRadius | 2 | Group | Circle | Contrast | 4.479 |
| F338 | MinFeretDiameter | 2 | ROI | Circle | Contrast | 3.992 |
| F732 | Entropy_max | 3 | Case | Nearest | Contrast | 3.737 |
| F424 | Perimeter | 2 | Group | Circle | Contrast | 3.587 |
| F311 | MedianRadius | 2 | Group | Circle | Contrast | 3.521 |

**Abbreviation,**

**Feature group 1 is nuclei average of standard deviation of ROI**

**Feature group 2 is nucleus shape related CFLCM heterogeneity feature**

**Feature group 3 is intra nucleus texture related CFLCM heterogeneity feature**

**A: Feature's number in analysis, B: Features name from CellProfiler, C: Group of feature name on line B, D: CFLCM calculation base**

**E: CFLCM neighborhood selection method, F: Haralick function, G: Weight on SVM model**

**Cell Profiler features:** http://cellprofiler-manual.s3.amazonaws.com/CellProfiler-3.0.0/modules/measurement.html

**CFLCM Method: Reference [14]**

**NI: no information**

(B) **Top 20 of highly contributed features to non-Recurrence**

| A | B | C | D | E | F | G |
|---|---|---|---|---|---|---|
| Feature No. | Feature Name | Feature group | Method | Neighborhood | Haralick Feature | Weight |
| F136 | FormFactor | 2 | Group | Nearest | Contrast | -13.061 |
| F141 | FormFactor | 2 | Case | Nearest | Contrast | -10.837 |
| F562 | AngularSecondMoment_max | 3 | ROI | Circle | Contrast | -9.272 |
| F456 | Solidity | 2 | Group | Nearest | Contrast | -9.128 |
| F151 | FormFactor | 2 | Group | Circle | Contrast | -8.676 |
| F904 | SumVariance_max | 3 | Group | Nearest | Contrast | -6.845 |
| F099 | Extent | 2 | ROI | Nearest | Contrast | -6.104 |
| F572 | AngularSecondMoment_max | 3 | Case | Circle | Contrast | -5.907 |
| F919 | SumVariance_max | 3 | Group | Circle | Contrast | -5.67 |
| F156 | FormFactor | 2 | Case | Circle | Contrast | -5.291 |
| F082 | Eccentricity | 2 | ROI | Circle | Contrast | -5.275 |
| F840 | SumAverage_max | 3 | Group | Nearest | Contrast | -5.2 |
| F200 | MaxFeretDiameter | 2 | Group | Nearest | Contrast | -4.994 |
| F557 | AngularSecondMoment_max | 3 | Case | Nearest | Contrast | -4.713 |
| F215 | MaxFeretDiameter | 2 | Group | Circle | Contrast | -4.478 |
| F584 | Contrast_max | 3 | Group | Nearest | Contrast | -4.186 |
| F508 | FracAtD_ratio | 2 | Case | Circle | Contrast | -4.172 |
| F195 | MaxFeretDiameter | 2 | ROI | Nearest | Contrast | -4.142 |
| F658 | DifferenceEntropy_max | 3 | ROI | Circle | Contrast | -4.106 |
| F471 | Solidity | 2 | Group | Circle | Contrast | -4.075 |

**Supplementary Table 4. Results of out of bag (OOB) error in RF model**

| **OOB estimate of error rate : 15.6%** | | | | |
|---|---|---|---|---|
| Confusion matrix | | | **Prediction** | |
| accuracy: 84.4% | | Rec (+) | Rec (−) | Total |
| | Rec (+) | 262 | 66 | 328 |
| **Truth** | Rec (−) | 43 | 327 | 370 |
| | Total | 305 | 393 | 698 |

Abbreviations: Rec, recurrence within 2-years

## Supplementary Figure 1. Segmentation mask creation of the touching nuclei

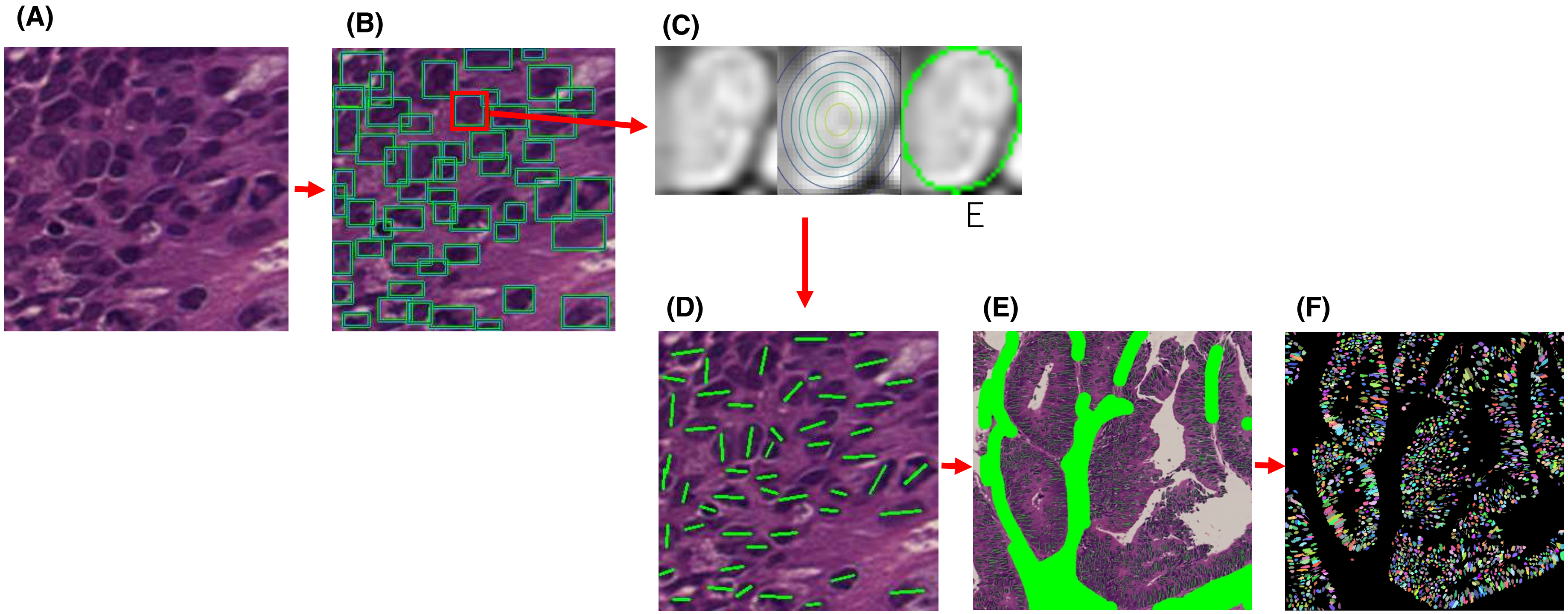


(A) original HE image (B) nucleus shape and area were detected using trained YOLO v3 model. (C) The nucleus image was changed to black-and-white, and the region was concentrically expanded from the center of the nucleus to extract the nucleus contour line. (D) and (E) the image showed nucleus maximum axis as process intermediate images, to check if individual nuclei were recognized, (F) a new additional segmentation mask. To construct segmentation process as a general-purpose process, we used colorectal cancer images for learning, because which is the highest cellularity tissue among cancer.

**Supplementary Figure 2. An illustration of the machine learning application in the clinical management of non-muscle invasive bladder cancer**

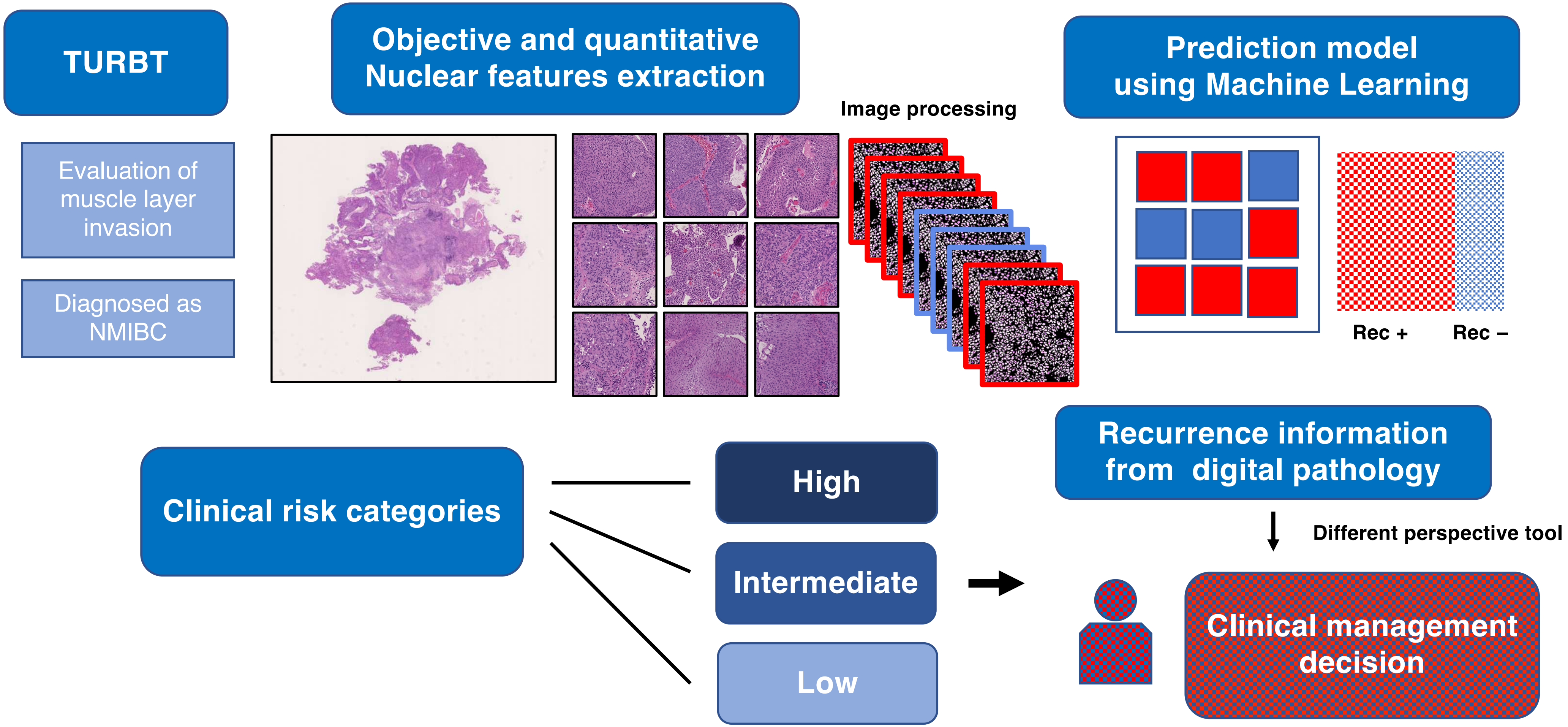

Supplement: Supplementary file 1 — Supplementary Material [file 41379_2021_955_MOESM1_ESM.pdf]
